# Preserving the Ephemeral: A Micro-Invasive Study on a Set of Polyurethane Scenic Objects from the 1960s and 1970s

**DOI:** 10.3390/polym15092111

**Published:** 2023-04-28

**Authors:** Rosa Costantini, Luca Nodari, Jacopo La Nasa, Francesca Modugno, Lucia Bonasera, Sara Rago, Alfonso Zoleo, Stefano Legnaioli, Patrizia Tomasin

**Affiliations:** 1 Institute of Condensed Matter Chemistry and Technologies for Energy, National Research Council, (ICMATE-CNR), Corso Stati Uniti 4, 35127 Padova, Italy; rosa.costantini@icmate.cnr.it (R.C.); patrizia.tomasin@cnr.it (P.T.); 2 Department of Chemistry and Industrial Chemistry, University of Pisa, Via Giuseppe Moruzzi 13, 56124 Pisa, Italy; jacopo.lanasa@unipi.it (J.L.N.); francesca.modugno@unipi.it (F.M.); l.bonasera@studenti.unipi.it (L.B.); 3 Department of Cultural Heritage: Archaeology and History of Art, Cinema and Music, University of Padova, Piazza Capitaniato 7, 35139 Padova, Italy; sara.rago@studenti.unipd.it; 4 Department of Chemical Sciences, University of Padova, Via Marzolo 1, 35131 Padova, Italy; alfonso.zoleo@unipd.it; 5 Institute of Chemistry of Organometallic Compounds, National Research Council, (ICCOM-CNR), Via Moruzzi 1, 56124 Pisa, Italy

**Keywords:** polyurethane foam, μ-ATR-FTIR, Py(cryo)-GC/MS, EGA-MS, performing artworks

## Abstract

Among the innovative materials used by 20th-century artists, polyurethane (PUR) has been shown to be highly unstable, and therefore artworks made of it are now in need of careful conservation strategies. This study presents a multi-analytical investigation of PUR foam scenic objects originally made between the 1960s and 1970s during the Italian Arte Viva movement. The main components in the foam and additives were characterized through micro attenuated total reflectance infrared spectroscopy (μ-ATR-FTIR) and pyrolysis coupled with gas chromatography and mass spectrometry (Py-GC/MS). Painted samples were further investigated through μ-FTIR and Raman spectroscopy to define binders and pigments. The use of μ-ATR-FTIR in combination with evolved gas analysis-mass spectrometry (EGA-MS) allowed the variable conditions of the artworks to be assessed and attained some insights into the chemical processes responsible for aging. At the same time, morphological changes due to the degradation phenomena were recorded through optical (OM) and scanning electron microscopy (SEM). The detailed characterization of the PUR foam and painting materials was helpful in attaining some insights into harmful environmental parameters for the artworks, thus informing preventive conservation.

## 1. Introduction

During the 20th century, a wide range of materials was synthesized and made commercially available, offering many artists the chance to experiment with them. Long-lasting rules on which products to use (e.g., supports, binders, pigments) and how to use them started not to apply anymore. At the same time, artwork’s durability, as well as the need to document the manufacturing approach, were no longer regarded as fundamental. Due to this, today, the characterization of contemporary art plays a critical role in the attempt to define artistic methods and trends better while addressing present and future conservation needs [1,2,3,4].

Among others, this experimental approach was at the basis of the Italian Arte Viva movement, which was born in the 1960s and grouped different artists, including Miela Reina. Miela Reina (Trieste, 1935—Udine, 1972) was an eclectic artist who partly devoted herself to creating theatrical performances. In addition to being a director and actress, Reina took care of the scenic design of her performances using, among other materials, polyurethane (PUR, whose general formula is -[-O-C(=O)NH-R-NHC(=O)-O-R’-]_n_-, R: aromatic or aliphatic group; R’: polyol ether, ET, or ester, ES) foam [5]. Back then, in the late 1960s, PUR (first synthesized in 1937) was becoming popular among designers and artists. Today, this raises several conservation issues since objects made of PUR have been shown to degrade within a few decades [1]. Especially when it comes to performing artworks, deciding if (and how to) conserve them is far from straightforward, as just defining their identity and interpreting the artists’ intentions can be challenging [6]. Even when opting for conservation, in the case of highly unstable PUR objects, methods and materials may differ, and some need more in-depth evaluation [1,7,8,9,10].

While ideal conservation approaches are still under study due to the versatility and widespread use of PUR, their degradation processes have been investigated by a number of researchers in various fields [11]. In general, hydrolysis and photo-oxidation are recognized as the two main mechanisms affecting PUR. In the case of foam, the material deteriorates even more rapidly than other types of PUR since the greater surface area eases the interaction (and reaction) with light, oxygen, and humidity [12,13]. Flexible PUR foams are complex polymers tightly self-associated through intramolecular hydrogen bonding [14]. The material is structurally composed of soft segments (polyols flexible chains, SS) and hard segments (mainly aromatic rings, HS) covalently bonded to each other. HS units are bonded through two types of crosslinks: urea linkage and hydrogen bonding, which gives rise to the physical crosslink of the PUR foam [14,15,16].

Depending on the main components (i.e., polyols and diisocyanate) and additives in the foam, PUR may age differently. Namely, ether-based PUR (PUR/ET) foam has been found to mainly undergo photo-oxidation processes due to the high sensitivity of polyether soft segments [17]. This causes the cleavage of bonds in the polymeric network and, eventually, the crumbling of the material. The presence of aromatic diisocyanates can also trigger photo-oxidation, first causing discoloration and ultimately leading to the formation of quinoid chromophores and, thus, yellowing [18,19]. Ester-based PUR (PUR/ES) foam is stated to be more prone to hydrolysis, though the stability is reported to be linked to the type of polyester [7]. As in the case of photo-oxidation, after hydrolysis, the molecular weight of the polymeric chains decreases, and this affects the mechanical properties of the material, such as flexibility and tensile strength [20,21,22].

Since museum objects made of PUR may show a different response to environmental parameters depending on their components, chemically characterizing the foam is critical for planning preventive conservation strategies [23]. To accomplish this, attenuated total reflectance infrared spectroscopy (ATR-FTIR) is often employed to discriminate between ether- and ester-based PUR, considering as diagnostic the intensity of the peaks from the stretching of the ether C-O-C group at 1100 cm^−1^ and from the ester O=C-O-C group at 1124 cm^−1^, respectively [7,14,24,25,26]. In addition, the technique has been shown to be useful in assessing the conservation state of PUR.

In addition to ATR-FTIR, artworks made of PUR have been characterized by analytical pyrolysis coupled with gas chromatography and mass spectrometry (Py-GC/MS) [12,26,27,28,29]. In addition to the type of polyol, the mass spectrometric approach permits the identification of specific monomeric precursors and additives, such as plasticizers [30]. On the other hand, the investigation by evolved gas analysis-mass spectrometry (EGA-MS) has provided meaningful insights into PUR degradation [31] and hence into the state of conservation of artworks [26,28,32].

While degradation mechanisms can be better described through the cited approaches, studying the morphology of the foam cells through optical microscopy (OM) and scanning electron microscopy (SEM) still represents a valuable option for assessing the condition of the polymeric network [7,14].

The present work aims to characterize a set of PUR foam objects from Miela Reina’s performances. Some of the case studies were created by the artist herself in the late 1960s, while others are replicas made by her colleagues Enzo Cogno and Carlo de Incontrera in the 1970s and 2020, respectively. For their characterization, μ-FTIR, μ-ATR-FTIR spectroscopy, and Py-GC/MS were employed to identify the type of polyol, additives, and binding media in samples from the painted PUR constituting the artworks, while Raman spectroscopy was used for pigments. Data from both μ-ATR-FTIR spectroscopy and EGA-MS were combined to assess the conditions of the objects, together with morphological information gathered from OM and SEM analysis. Comparing data collected from the invasive spectroscopic approach and destructive EGA-MS allowed discussing further the usefulness of each technique in defining the state of conservation of PUR museum objects. The various dating of the samples was instrumental in validating the analytical methods. Ultimately, the work intends to provide helpful pieces of information for the future preventive conservation of the PUR artworks studied.

## 2. Materials and Methods

### 2.1. Artworks and Collected Samples

**Figure 1:**
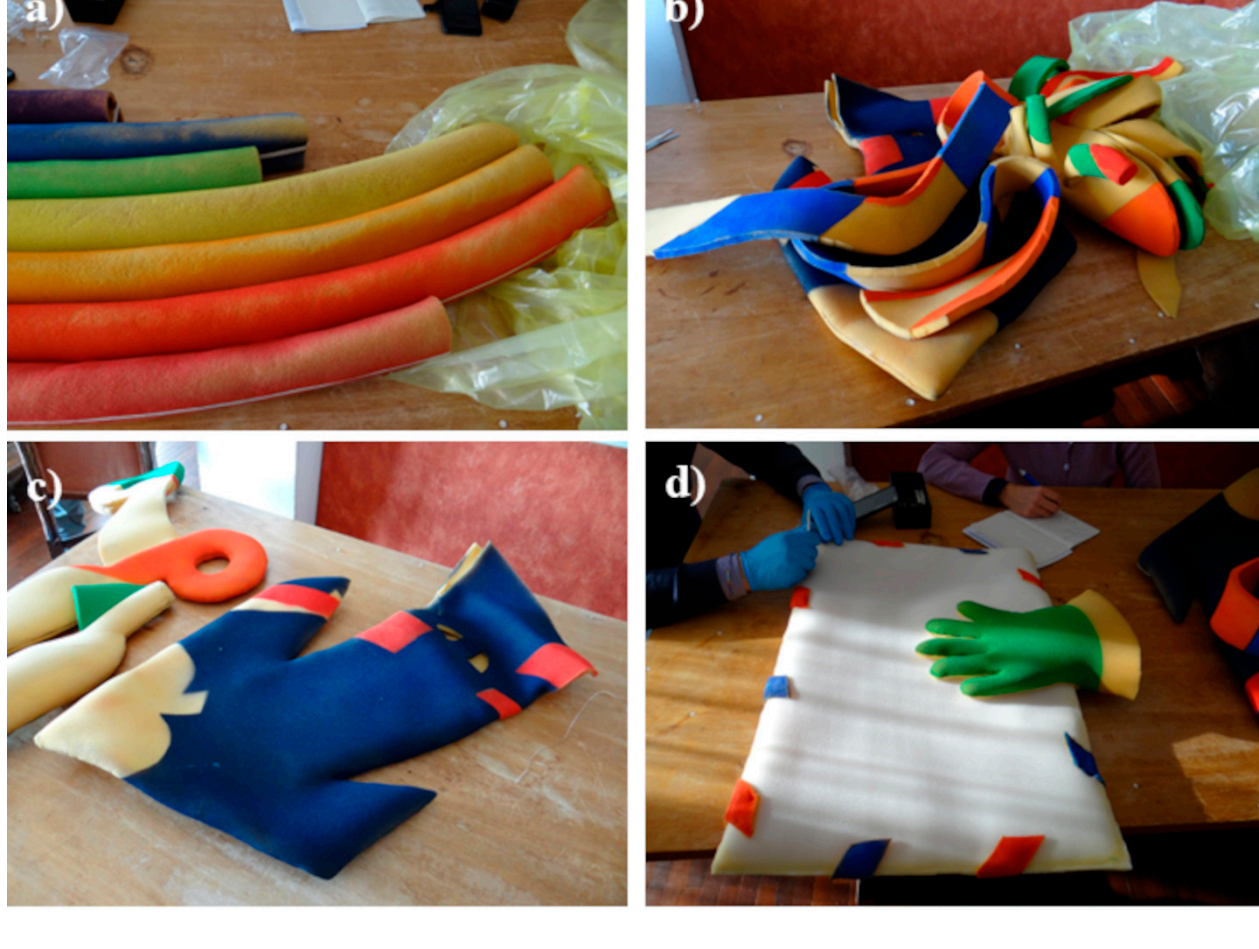
Some of the studied artworks: (**a**) *Rainbow* by Miela Reina; (**b**) *Dragon*, (**c**) *Scissors* and *Arrow* by Enzo Cogno; (**d**) *Envelope* by Carlo de Incontrera.

In total, ten PUR foam artworks were studied. As reported by de Incontrera, when manufactured, they were colored through spray painting. For each of the scenic objects, a few samples were collected from different representative areas (e.g., painted/unpainted, glued/unglued). Table 1 presents the list of artworks and samples analyzed through different analytical techniques. The dating of the artworks is reported in Table 1, together with their author. Figure 1 shows pictures of some of the artworks taken during the sampling campaign. The images clearly depict the 3D structure of the objects, involving the bending and folding of the PUR foam. This, combined with the fact that the artworks are scenic objects originally intended to be worn and manipulated, may be responsible for the mechanical degradation of the supporting and painting materials. It is noted that physical degradation phenomena are not discussed in the current paper, which focuses on assessing the state of conservation mainly from a chemical perspective.

In addition to the samples taken from the scenic objects, a reference PUR sample was also investigated. This material was provided by de Incontrera, who stated it should be the same PUR foam that he employed to reintegrate the collection in 2020, when indeed he remade the object *Envelope*. From this perspective, we can consider this sample as a reference, as it represents the unpainted supporting material and is the least subject to degradation. Considering the aim of the study, investigating the reference material was instrumental in defining whether there is any difference in the conditions of the objects that can be related to their composition and evaluating the effectiveness of the selected analytical approaches in revealing such differences.

### 2.2. Analytical Techniques

Micro-infrared spectroscopy measurements were carried out using a Thermo Fisher Nicolet is10 spectrometer combined with a Continuum microscope equipped with an MCT (mercury-cadmium-telluride) detector. Spectra were collected in micro-ATR and/or micro-reflectance mode. For μ-ATR-FTIR analysis, a Si crystal was mounted on a Cassagrain objective. The spectra were collected in the 4000–650 cm^−1^ range, accumulating at least 128 scans with a resolution of 4 cm^−1^. Prior to micro reflectance analyses, painted PUR samples were treated, on the high reflectivity glass slide, with a few drops of CH_3_Cl, (Aldrich spectroscopic grade) to separate the paints from the PUR support.

Morphological investigations were performed by optical (OM) and electronic microscopy (SEM). OM images were obtained by means of an Olympus SZX12 (Olympus Corp. Tokyo, Japan) equipped with a digital camera, while SEM analysis was conducted using an FEI Quanta 200F equipped with a field emission gun source. SEM images were acquired by collecting secondary electrons using an Everhart–Thornley detector (HV: 5 kV, working distance 8–10 mm, spot size 5.4–5.5).

Raman analysis was carried out on a Renishaw InVia confocal instrument coupled with an optical Leica DLML microscope equipped with an NPLAN 50× objective. Two laser sources were used, maintaining the laser power below 0.5 mW: a He-Ne laser (λ = 633 nm); a diode laser (λ = 785 nm). The spectrometer consists of a single grating monochromator (1800 lines mm^−1^ for the He-Ne laser, 1200 lines mm^−1^ for the diode laser) coupled with a CCD. Spectra were acquired in the range between 100 and 3200 cm^−1^ with a resolution of 0.5 cm^−1^ @ 633 nm and 2 cm^−1^ @ 785 nm. The spectral calibration of the instrument was performed on the 520.0 cm^−1^ band of a pure silicon crystal.

For both Py(cryo)-GC/MS and EGA-MS analyses, the samples (whose weight ranged between 100 and 200 μg) were placed into a pyrolysis stainless-steel cup. Then, they were inserted in a microfurnace Multi-Shot Pyrolyzer EGA/Py-3030D (Frontier Lab, Fukushima, Japan) coupled with an 8890 gas chromatograph, combined with a 5977B mass selective single quadrupole mass spectrometer detector (Agilent Technologies, Santa Clara, CA, USA).

For Py(cryo)-GC/MS measurement, GC injector temperature was set at 280 °C and injection was operated in split mode (split ratio of 1:10). The cryo focus experiments were performed using a MicroJet Cryo-Trap MJT-1035E (Frontier Lab, Fukushima, Japan), with a focusing time of 2 min. The chromatographic separation of pyrolysis products was performed on a fused silica capillary column HP-5MS (5% diphenyl-95% dimethylpolysiloxane, 30 m × 0.25 mm i.d., 0.25 μm film thickness, J&W Scientific, Agilent Technologies), preceded by 2 m of deactivated fused silica pre-column with an internal diameter of 0.32 mm. The chromatographic conditions were: 36 °C for 10 min and then from 10 °C/min to 310 °C for 20 min. The helium (purity 99.9995%) gas flow was set in constant flow mode at 1.2 mL/min. MS parameters: electron impact ionization (EI, 70 eV) in positive mode; ion source temperature 230 °C; scan range 50–700 *m*/*z*; interface temperature 280 °C. Perfluorotributylamine (PFTBA) was used for mass spectrometer tuning. MSD ChemStation (Agilent Technologies) software was used for data analysis, and the peak assignment was based on a comparison with libraries of mass spectra (NIST 20) and the literature data.

In the case of EGA-MS, the PY and MS system was interfaced with an Ultra ALLOY ^®^ EGA Tube (2.5 m × 0.15 mm i.d). The following temperature program was selected for the microfurnace chamber of the pyrolyzer: initial temperature 50 °C; 10 °C/min up to 800 °C for 10 min. Analyses were performed under a helium flow (1 mL/min) with a split ratio of 1:20. The microfurnace interface temperature was automatically kept at 100 °C higher than the furnace temperature up to the maximum value of 300 °C. The inlet temperature was 280 °C. The mass spectrometer was operated in EI positive mode (70 eV, scanning *m*/*z* 50–700). The MS transfer line temperature was 300 °C. The MS ion source temperature was kept at 230 °C, and the MS quadrupole temperature was 150 °C.

## 3. Results and Discussion

### 3.1. Characterization of the Samples

At first, PUR and painting materials constituting the samples were characterized using the selected invasive (μ-FTIR, μ-ATR-FTIR, Raman spectroscopy) and destructive (Py-GC/MS) analytical methods.

#### 3.1.1. PUR Foam

The μ-ATR-FTIR analysis carried out on unpainted samples from different objects allowed us to characterize the foam as PUR/ET. Indeed, the spectra collected on the unpainted portion of the samples show, in addition to the other typical PUR signals, the presence of the intense peak at ≈1100 cm^−1^ attributable to ν_s_(C-O-C) in ether-based polyol [14,26]. The use of aromatic isocyanates as precursors can be deduced from the distinct absorption, at 1599 cm^−1^, due to ν(C=C) in the benzene ring, together with the weak but well-resolved ones at 865, 815, 758 cm^−1^ from the out-of-plane wagging and rocking vibrations of the C-H in benzene rings [18]. The spectrum of the reference material and its attributions are reported in supplementary materials (in Figure S1 and Table S1, respectively). In general, all the spectra show the signals attributable to the H-bond linkage between the HS units of the polymer. These distinctive absorptions are the ν(N-H) at 3289 cm^−1^ and the ν_a_(C=O) at 1641 cm^−1^, ascribable to strongly H-bonded bidentate urea. It is relevant to observe that all the spectra collected show features indicating PUR degradation; these aspects will be discussed in detail in Section 3.2.2.

**Figure 2:**
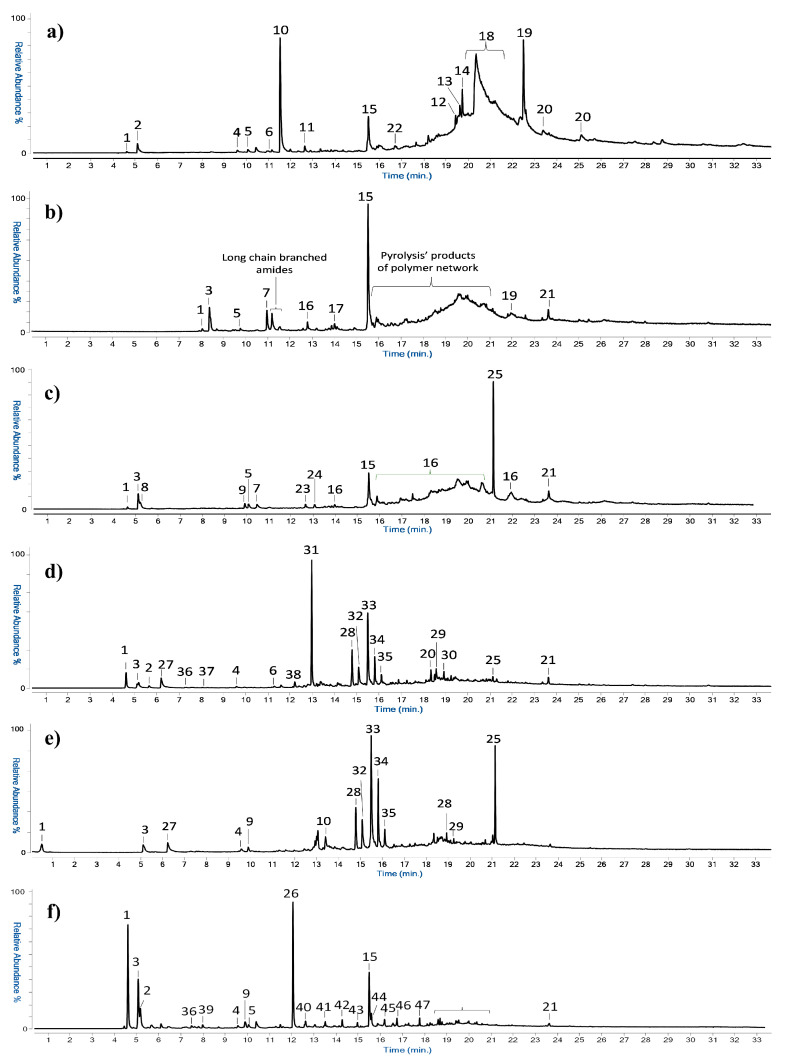
Py-GC/MS chromatograms obtained for the samples (**a**) REF; (**b**) TR_1; (**c**) PAR_1; (**d**) AR_3; (**e**) BVC_3; (**f**) ENV_2.

The IR characterization of the polyurethane polymer and the identification of the type of polyol is in agreement with the Py(cryo)-GC/MS results. Table S2 shows the compounds identified by mass spectrometry in the pyrolytic profiles of the different samples, with the respective *m*/*z* ions, while Figure 2 reports the GC/MS chromatograms obtained by pyrolysis for six different samples.

The Py-GC-MS chromatogram of the reference material (Figure 2a) features the pyrolytic markers of PUR polymer: 2,4- and 2,6-diisocyanotoluene, with mass spectra characterized by the main ions *m*/*z* 174, 145, 132, 118, 91, 76. In the chromatogram of sample TR_1 (Figure 2b) and PAR_1 (Figure 2c), the presence of the polyols tripropylene glycol (*m*/*z* 103 and 59) and dipropylene glycol confirms the use of a PUR/ET foam. In the case of sample TR_1, the pyrolytic profile shows the presence of long-chain amides at retention times between 11 and 12 min and a series of pyrolysis products related to the polymeric network. Even if it was not possible to achieve an optimal chromatographic separation for these species, these pyrolysis products of the network could be identified as ether-based chemical species, supporting the results obtained in the μ-ATR-FTIR characterization.

In addition to confirming the use of PUR/ET, Py(cryo)-GC/MS analysis of the REF sample revealed a series of peaks associated with the pyrolysis of an acrylonitrile-styrene based copolymer (peaks n. 4, 10–14 of Table S2). This material is mostly used in applications where better chemical and temperature resistance than standard PUR foams is required. The group of peaks identified between 16 and 20 min is characteristic of erucamide and includes a series of its branched isomers whose chromatographic separation was not possible. These chemical species are used as additives in plastic manufacturing. Together with them, the analysis highlighted the presence of the plasticizer diethyl phthalate. The same plasticizer was also identified in the samples from the analyzed works of art (*Parachutist*, *Rainbow*, *Big Vibrating Character*, *Glove*, *Arrow*, and *Envelope*), with the only exception of sample ENV_1.

In general, it is noted that Py(cryo)-GC-MS chromatograms obtained for most of the samples from *Parachutist*, *Rainbow*, *Big Vibrating Character*, and *Glove* (with the exception of GL_2, as discussed in Section 3.1.2) show similar pyrolytic profiles. In particular, the first part of the chromatogram presents low molecular weight compounds deriving from the pyrolysis process of the polymeric network of the PUR foam, while at higher retention times, there are peaks associated with the alcohols used in the polymer synthesis and the phthalate plasticizers. Only in sample AR_3 (Figure 2d) butylhydroxytoluene, an antioxidant additive, was also observed. In the case of samples AR_3 and BVC_3, compounds related to the binding media were detected, and this is discussed in detail in the following Section. The analysis detected, in different samples, squalene and cholesterol deriving from the skin of the hands. This is likely due to contamination during sampling and/or previous handling of the artworks.

#### 3.1.2. Binding Media

**Figure 3:**
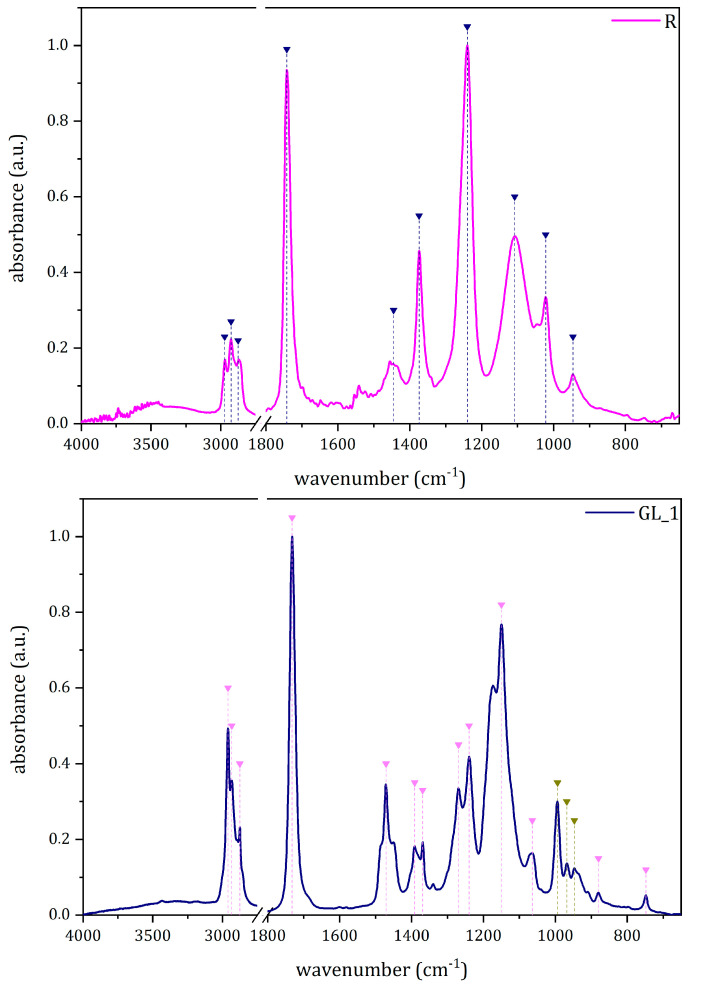
The μ-FTIR spectra of the identified binders: PVAc (**Top**) and acryl binders (**Bottom**). Dark blue and pink triangles indicate the position of PVAc and acryl references, respectively. Dark yellow triangles stand for the iso-butyl methacrylate markers.

After separating the paint layer from the PUR substrate with a few drops of CHCl_3_, painted samples were analyzed through μ-FTIR spectroscopy. The approach allowed distinguishing two binding media among the different samples, polyvinyl acetate (PVAc) and acrylic. For example, Figure 3 depicts the IR spectra collected from samples R_1 and GL_2 and the characteristic peaks attributed to the binding media. In general, all the acquired spectra present some common signals, such as those from the C=O asymmetric stretching (at around 1736–1725 cm^−1^, depending on the polymer) and those from the C-H stretching between 2965 and 2865 cm^−1^. All the samples taken from *Rainbow* (e.g., R_1, Figure 3, top), as well as the red ones from *Tree* (TR_1) and *Glove* (GL_1), showed the diagnostic peaks of PVAc centered at around 1240 cm^−1^ (C-O stretching) and 1373 cm^−1^ (C-H_3_ bending) [33,34,35]. While the identification of PVAc was straightforward in the case of *Rainbow* samples, it should be noted that data from TR_1 and GL_1 show some spectral differences in the peaks at 1640 cm^−1^ and 1540 cm^−1^. As discussed in Section 3.1.1, such signals are likely related to the PUR substrate. For the blue *Glove* sample (GL_2), the IR spectrum (Figure 3, bottom) clearly suggests the use of an acrylic binder, likely containing iso-butyl methacrylate. The identification is based on the characteristic peaks at 1240 cm^−1^ from C-C and C-O stretching, 1470 cm^−1^ from methylene bending, and 1150 cm^−1^ from the ester groups C-O-C stretching [36,37,38,39]. Although it is difficult to define the specific type of acrylic resin, the triplet at 994, 968, and 947 cm^−1^ may indicate the presence of iso-butyl methacrylate [40], as later confirmed by Py(cryo)-GC/MS.

The characterization of the binding media was confirmed and integrated by the Py(cryo)-GC/MS analysis. In particular, the use of an acrylic resin in sample GL_2, as well as in sample ENV_2, was identified on the basis of the high abundance of iso-butyl methacrylate, as reported in the chromatograms of Figure 2f. On the other hand, as shown by the results in Figure 2d,e, PVAc was found in samples AR_3 and BVC_3 from the artworks *Arrow* and *Big Vibrating Character*, respectively. PVAc was detected in the samples (also showing the characteristic peaks of the PUR polymer, as previously discussed) thanks to the presence of acetic acid, benzene, and several aromatic pyrolysis products [26,27,41]. The results from both μ-FTIR and Py(cryo)-GC/MS analyses are summarized in Table S3.

In addition to the binding media, chloroprene-based rubber was revealed in samples AR_3 and BVC_3. The presence of the rubber was assessed by the identification of chloroprene together with 1-chloro-4-(1-chlorovinyl) cyclohexene and high molecular weight pyrolysis products specific to this material. A different type of rubber was found in sample ENV_2 where the detection of peaks 39–47 (Table S2), with increasing molecular weight characterized by the common principal ions *m*/*z* 125, 111, 97, 83, 70, 55, allowed to assess the presence of EPDM rubber (Ethylene-Propylene Diene Monomer). In general, the pyrolytic profiles of these rubbers are characterized by the presence of unbranched long-chain alkenes with increasing carbon numbers. The rubbers found in some of the samples from the three artworks, *Arrow*, *Big Vibrating Character*, and *Envelope*, could be related to the use of an adhesive for assembling the scenic objects. It is important to observe that IR spectroscopy did not allow the identification of any adhesive. As a matter of fact, the spectra collected on these samples were dominated by the absorptions due to the substrate and/or the binders. Moreover, in the case of *Envelope*, no adhesive was visually observed prior to the analysis, though its presence is consistent with the manufacturing technique of the scenic objects.

#### 3.1.3. Pigments

Raman measurements were performed on some selected samples from the artworks made by the three artists. Pigments were identified by comparing the acquired spectra (Figure S2) with spectral data reported in libraries and the literature [42,43].

In general, the results show the presence of both modern organic and inorganic pigments, typical of commercial formulations available during the manufacturing period of the artworks [44]. The results are summarized in Table S4. In almost all the samples, Raman measurements suggest a single pigment formulation; indeed, only sample R_7 shows the presence of a mixture of different pigments. In particular, the dark orange hue of the paint in R_7 is obtained by the combination of yellow PY83 and red PR112. In the case of sample TR_1, pigment identification was not straightforward, as the presence of bands associated with PUR components tends to mask the pigment peaks, which can be ascribed to signals of a quinacridone polycyclic pigment.

### 3.2. Assessment of the Conditions of the PUR Foam

After the materials characterization, insights into the state of conservation of the samples and the degradation mechanisms potentially affecting them were gathered from microscopy, μ-ATR-FTIR, and EGA-MS analysis.

#### 3.2.1. OM and SEM

**Figure 4:**
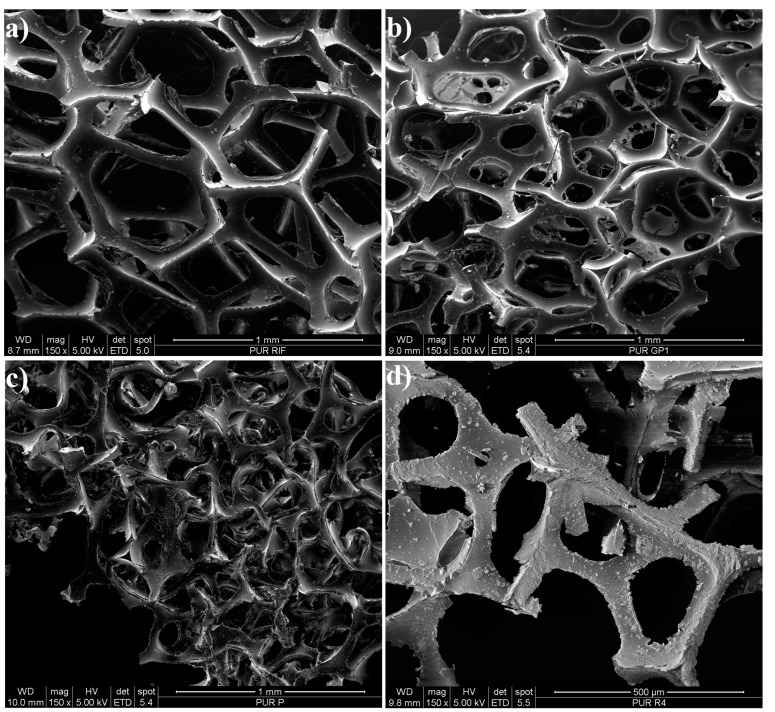
Secondary electrons SEM images of samples from REF (**a**); *Big Vibrating Character* (**b**); *Parachutist* (**c**); *Rainbow* (**d**).

Before µ-ATR-FTIR and EGA-MS measurements, the morphology of PUR unpainted samples was studied through OM (see Figure S3) and SEM analysis (see Figure 4 and Figure S4). In Figure 4, magnified pictures of some representative samples are provided. From the comparison of the images, irregularities of the structure and texture of the PUR foam from the artworks are evident, though differences in the type of heterogeneities can be distinguished from sample to sample. In particular, when looking at the SEM images of PAR_1 (Figure 4c), the extensive failure of the 3D network characteristic of PUR foam can be noted. Moreover, pitting is widespread all over the surface. In sample R4, depicted in Figure 4d, the numerous cracks along the perimeter of the foam skeleton state the poor conditions of the material. On the other hand, in the case of the REF sample (Figure 4a), the alveolar structure and the borders appear to be less compromised, though some holes can still be observed on the surface.

It is interesting to note that, while in naturally aged samples, the effects of degradation are clear from superficial and structural heterogeneities, which appear consistent throughout each sample, the thickness of the cells struts shows to be quite variable even within the same sample. Due to this, employing the strut width measurement method suggested by Van Oosten [7] for defining PUR conditions is not straightforward. Nonetheless, as reported in Table S5, it can be observed that in the REF sample, the average cell strut width is 71 μm, while in the analyzed original samples from the 1960s and 1970s, it is between 62 and 55 μm (PAR_1 and R_1 showing the lowest values, 55 μm). According to Van Oosten’s criteria [7], the conditions of REF can be classified as poor and indicate the need for consolidation treatment. On the other hand, the artworks’ samples can be defined in bad conditions and thus require highly urgent consolidation.

#### 3.2.2. μ-ATR-FTIR

**Figure 5:**
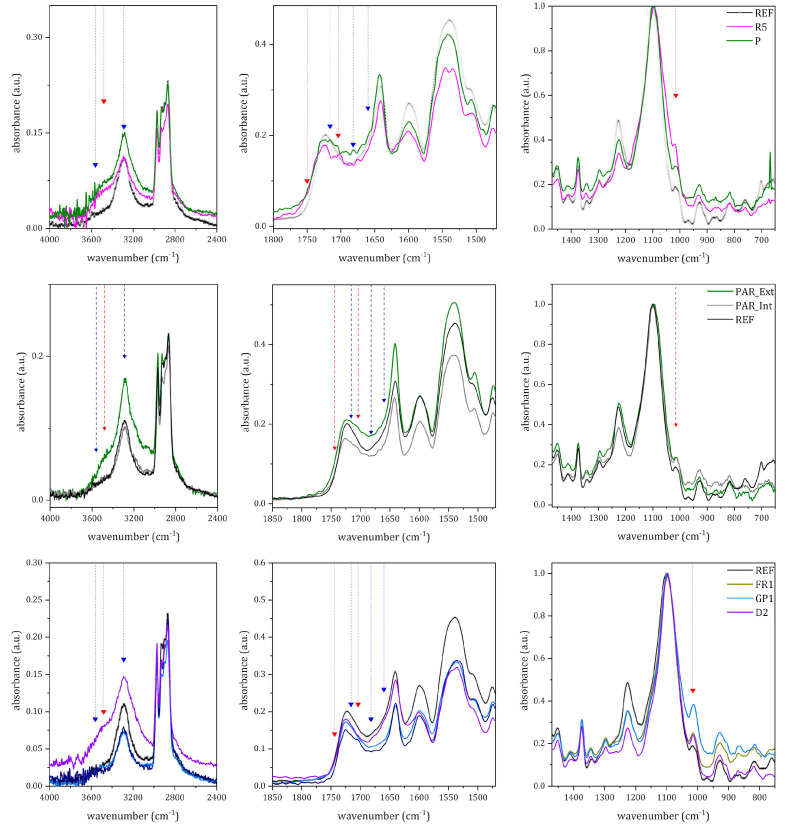
Comparison between the μ-ATR spectra collected on REF (black line) and the ones registered on: *Rainbow* and *Parachutist* (**top**); outer and inner surface of *Parachutist* (**middle**); *Dragon, Big Vibrating Character* and *Arrow* (**bottom**). Red arrows highlight the insurgence of absorption due to the modification of the soft segments, and blue lines are the ones involving the hard segments.

Spectral data gathered from the μ-ATR-FTIR analysis of the samples were further elaborated to define the state of conservation and degradation mechanisms affecting the PUR foam. Representative IR spectra of different samples are depicted in Figure 5.

By comparing the REF spectrum with the ones from the artworks’ samples, the main modifications are noted in the C=O stretching region. Another minor but significant variation concerns the absorptions due to H-bond linkages. The latter can be related to the decrease in linkages among the hard segments (i.e., aromatic rings bonded through urethane units) in PUR. As later better discussed, these differences can be noted not only between aged samples and the REF one, but also among samples from the original artworks spanning across two decades (the 1960s and 1970s) and among areas belonging to the same object (e.g., outer and inner surface in PAR).

As mentioned in Section 3.1.1, also REF spectrum shows some features describing an incipient alteration. Indeed, a careful analysis of the urethane ν_a_(C=O) peak, at 1723 cm^−1^, shows a shoulder at 1710 cm^−1^, suggesting the presence of urethane groups loosely associated through H-bond [14,26]. At the same time, the shoulder at 1660 cm^−1^ could be ascribed to ν(C=O) in monodentate urea. Moreover, the shoulder at 3360 cm^−1^, and the weak absorption at 1015 cm^−1^, can be respectively attributed to ν(O-H) and to ν(C-O-C) in alcohols and products of the photo-oxidation reactions [17].

The effects of aging are particularly evident in the samples from *Rainbow* and *Parachutist*, the oldest artworks studied. In these samples, there are remarkable spectral modifications in the 4000–3000 cm^−1^ region and between 1800 and 1600 cm^−1^. By comparing the spectra collected on PAR_1 and R_5 (Figure 5, top), it is clear that the stretching modes due to free N-H and O-H groups, at 3563 cm^−1^ and 3360 cm^−1^, increase in intensity. While the former is associated with the presence of free urea [26], the latter can be ascribed to the formation of photo-oxidation products and their subsequent hydrolyses, such as alcohols, acids, and hydroperoxyl [14]. The presence of these products is also supported by the distinctive shoulder at ≈1750 cm^−1^, together with a weak absorption at ≈1704 cm^−1^. These signals can be respectively related to the formation of ester and acids. Furthermore, in the fingerprint region, the intensity, in comparison to the REF spectrum, of the alcohol ν(C-O-C) at 1015 cm^−1^ confirms the occurrence of photo-oxidation reactions.

It is interesting to observe that the sample from *Parachutist* presents a more intense H-bond ν(N-H) signal at around 3290 cm^−1^ than the REF sample. This can be related to the formation of new chemical species, perhaps amines, as suggested by La Nasa et al. [21], or new N-H bonds involving the hard segments of PUR, as stated by França et al. [14].

The presence of free urea in PAR_1 is confirmed by the weak absorption at 1682 cm^−1^ (1677 cm^−1^ in R_5), attributable to the ν(C=O) in free urea as a consequence of the cleavage of urethane bond. Concerning the modifications of the hard segments, it should be noted that there is an increase in intensity in the abovementioned ν(C=O) signals at ≈1710 cm^−1^, ≈1660 cm^−1^, and ≈1650 cm^−1^. Interestingly, samples from *Parachutist* appear to show a different extent of alteration depending on the side. The spectra collected on the outer side, thus directly exposed to the environment, show a more intense H-bonded ν(N-H) peak than those from the inner part and REF (Figure 5, middle).

Similarly, though less remarkably than in *Parachutist*’s samples, some spectral modifications can be observed in Cogno’s artworks (Figure 5, bottom). Indeed, in Cogno’s samples (from the 1970s), signals related to free urea and variations of the hard segments are less evident. The only clear modification is the broadening towards the lower wavenumber of the ν(C=O) absorption at ≈1723 cm^−1^. Regarding other spectral regions, only spectra collected on D_2 show an increase in intensity in the free ν(N-H) at 3563 cm^−1^ and a non-well-resolved signal at 1682 cm^−1^ ascribable to free urea. It should be highlighted that the spectra collected on *Envelope*, the most recent object, do not present any remarkable difference from the one acquired on REF.

In summary, IR signals highlighting the occurrence of aging in PUR were related to the depolymerization of the foam (broadening of the signal at ≈1723 cm^−1^) and the formation of photo-oxidation products of lower molecular weight (an increase in signal intensity at 3563 cm^−1^, ≈3360 cm^−1^, ≈1710 cm^−1^, ≈1680 cm^−1^, 1660 cm^−1^, 1650 cm^−1^, 1015 cm^−1^).

#### 3.2.3. EGA-MS

**Figure 6:**
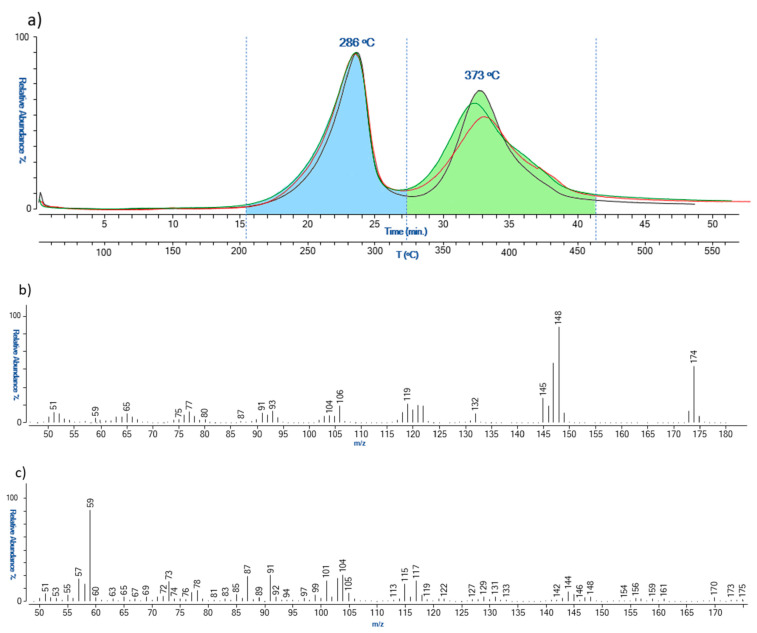
EGA-MS thermal profiles (**a**), and related mass spectra, (**b**,**c**), obtained for the reference polyurethane (REF).

Evolved gas analysis was carried out to characterize the sampled materials further and to evaluate the conditions of the PUR polymer on the basis of the different heating temperatures of desorbed/generated components. More specifically, this analytical approach enables us to assess the degree of depolymerization of PUR, as well as the possible formation of high molecular weight reticulation compounds. The reference material provided by de Incontrera was analyzed in triplicate first to evaluate the reproducibility of the temperatures of thermal degradation. The EGA-MS thermal profiles are reported in Figure 6.

The analysis showed the presence of two thermal degradation zones, the first one in the range of 200–320 °C and the second one in the range of 320–460 °C. Figure 6b reports the average mass spectra obtained for the two zones. The first thermal degradation zone is related to the desorption of non-crosslinked polymerization precursors with 2,4-diisocyanotoluene as the most abundant compound (*m*/*z* 174, 145, 132, 119, 106, 91, and 76). In the same zone, ion *m*/*z* 148, attributable to diisocyanotoluene amine derivatives, was revealed. It is noted that these chemical species can also be due to the partial thermal degradation of the lower molecular weight polymeric network. The second zone of the thermogram was instead characterized by the presence of the ions *m*/*z* 117, 104, 101, 91, 87, 73, 59, associated with the complete pyrolysis of the polymeric network.

**Figure 7:**
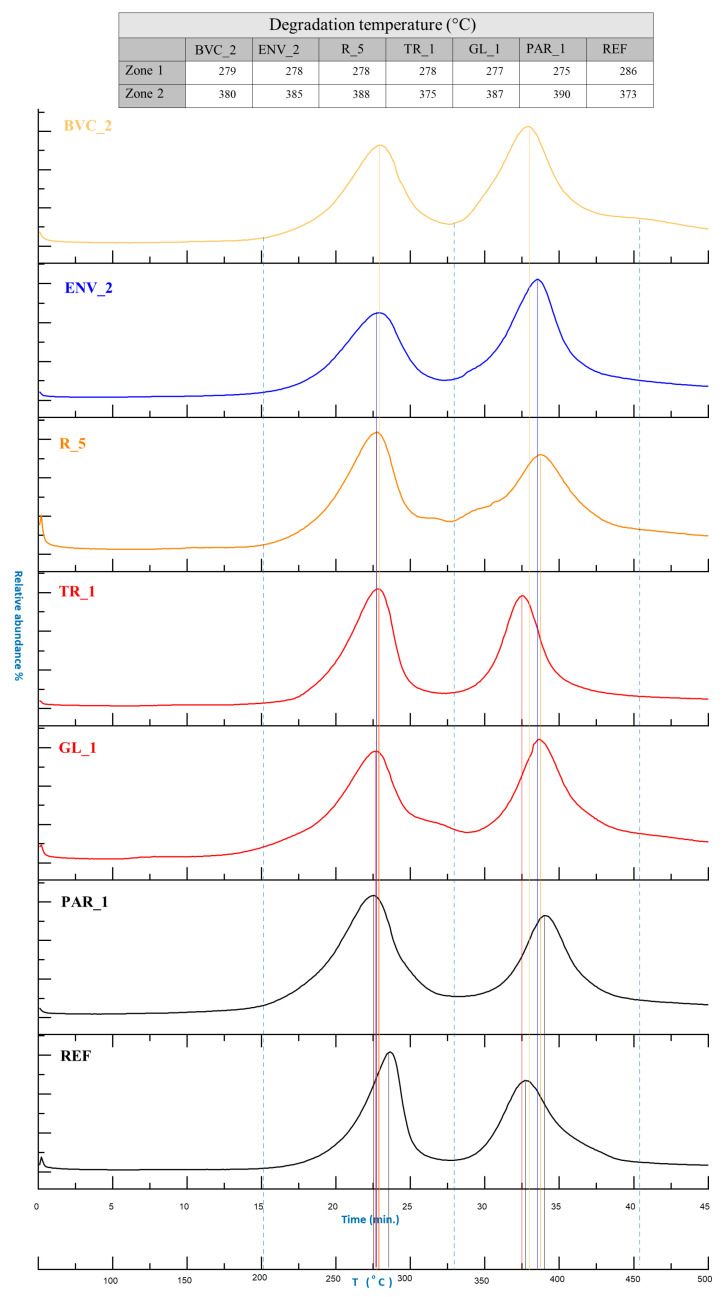
Thermal degradation profile of the different samples, as determined by EGA-MS.

In general, the thermograms obtained for the samples taken from the various artworks all show very similar thermal profiles (Figure 7). The first thermal degradation step (200–325 °C) presents ions associated with the thermal desorption of phthalate plasticizers, together with the non-crosslinked precursor and the chemical species deriving from partial degradation of the polyurethane polymeric network. On the other hand, the thermal degradation step in the range of 325–455 °C is due to the complete pyrolysis of the polymeric network.

By comparing the temperatures of the thermal degradation zones of the different samples, some observations on the conservation state can be drawn. Figure 7 reports the thermal profiles obtained for REF and the samples from the scenic objects. In general, a decrease in the temperature of the first thermal degradation step is indicative of the occurrence of hydrolysis processes, while an increase in the temperature of the second degradation step is associated with the formation of new reticulation products. This cross-linking phenomenon derives from the chemical interactions of the hydrolysis products, characterized by reactive functional groups, such as amino and hydroxyl groups. This indicates that there is a strict correlation between hydrolysis and reticulation processes occurring during the aging of PUR foams.

The thermograms highlighted that, in comparison to REF, the samples from works of art were all characterized by lower temperatures in the first thermal degradation step and higher temperatures in the second one. In particular, the samples from *Parachutist* and *Rainbow*, the oldest and visibly the most degraded among the studied artworks, presented the lowest and the highest temperatures for the two zones, respectively. It is pointed out that, on the basis of the temperature values, the PUR foam of ENV_2 (made in 2020) should be considered degraded as one of the samples from the 1960s. This outcome seems to disagree, not only with the dating of the artwork, but also partly with the magnified pictures and IR spectra acquired. Although it is not possible to combine and integrate all the different analytical observations, it should be noted that the representativeness of the sampling location and/or the use of specific manufacturing processes might have contributed to the contrasting data. Moreover, if comparing IR results with EGA-MS ones, it is underlined that the first technique only allows punctual investigation, while for the latter, the sample is analyzed in bulk.

**Figure 8:**
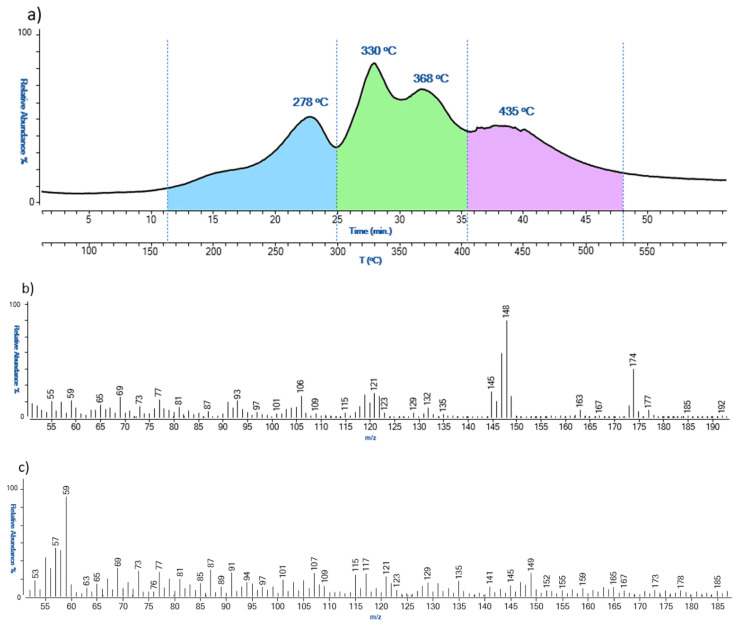
EGA-MS thermal profile (**a**), and related mass spectra (**b**,**c**), obtained for sample AR_3.

The EGA-MS profile obtained for sample AR_3 from *Arrow* was the only one with a completely different thermogram. The thermogram, as shown in Figure 8, presented three thermal degradation zones: zone 1 from 160 °C to 300 °C, zone 2 from 300 °C to 405 °C, and zone 3 from 405 °C to 530 °C. As observed for the other samples, zone 1 was characterized by ions associated with the desorption of non-crosslinked diisocyanates and the partial pyrolysis of the lower molecular weight polymeric network. On the other hand, in the second thermal zone, there are ions *m*/*z* 135, 121, 107, 91, and 77, diagnostic of butylhydroxytoluene (BHT), and the *m*/*z* 149 characteristic of phthalates. BHT is a lipophilic organic compound used in polymer formulations for its antioxidant properties.

In the last thermal degradation zone of the thermogram, ions deriving from the complete thermal degradation of the polymeric network were revealed. The degradation temperature of the second zone was characterized by a much higher temperature with respect to the other samples, suggesting that the type of PUR used for this artwork was characterized by a different crosslinking degree.

To sum up, the two thermal degradation zones that can be helpful for the assessment of the conditions of the artworks are between 200–325 °C (lower temperature, higher degradation); 325–455 °C (higher temperature, higher degradation).

## 4. Conclusions

The present work aimed to characterize and verify the state of conservation of some scenic objects originally made using polyurethane PUR foam between the 1960s and 1970s during the Italian Arte Viva contemporary movement. Moreover, the study intended to further evaluate the viability of some selected analytical techniques (OM, SEM, μ-ATR-FTIR, EGA-MS) for assessing the conditions of the artwork while describing related chemical degradation mechanisms.

The use of μ-ATR-FTIR spectroscopy and Py(cryo)-GC/MS determined that the objects are made of an ether-based PUR/ET foam. As assessed by μ-FTIR and Raman measurements on colored samples, the PUR foam of the artworks was painted using acrylic and polyvinyl acetate (PVAc) binding media, both containing typical pigments of commercial formulations of the time, e.g., phthalocyanines and monoazo pigments.

As expected from the fact that the foam is made of PUR/ET, μ-ATR-FTIR highlighted the occurrence of photo-oxidation degradation mechanisms (mainly evident in the region between around 1720–1650 cm^−1^) responsible for the rupture of the polymeric chains and the formation of lower weight chemical species, such as alcohols. At the same time, through the analysis of the thermal profiles, EGA-MS measurements showed that the material was also affected by hydrolysis. Combining the information gathered from μ-ATR-FTIR and EGA-MS, it can be deduced that hydrolysis reactions likely involved the chemical species formed through photo-oxidation, rather than the unaged PUR/ET polymer. The effects of such phenomena were clearly observed in the magnified pictures acquired through OM and SEM, which indicate various types of damage to the cells’ alveolar structure and texture (e.g., failure of the alveolar structure, cracks, and pitting). All the analytical methods for monitoring the state of conservation were used invasively and thus required the collection of samples. The details gathered through the non-destructive microscopic techniques were helpful in defining the state of conservation of the PUR foams. Nevertheless, they were not informative enough to understand degradation phenomena, which were described by μ-ATR-FTIR and EGA-MS. Indirectly, this confirmed the relevance and usefulness of the multi-analytical approach.

The outcomes of the three micro-invasive approaches were able to assess the non-optimal conditions of all the artworks, but in particular, they pointed out the poor state of conservation of the earliest objects made by Miela Reina in the 1960s, and hence the need for planning conservation strategies. When dealing with contemporary performing artworks, conservation strategy should take into consideration all the different needs/goals and the subjects involved. The strategy should aim at preserving the idea of the artist and the performance finality of the objects through the remaking of the deteriorated ones. At the same time, it is important to preserve the unicity and thus the materiality of the artworks made by the artists Miela Reina and Enzo Cogno (which can be considered original, too [45]), through a suitable “tailor-made” restoration and preventive conservation. The latter should be addressed by keeping the objects in dark conditions and avoiding extreme humidity, as indicated by the analytical results on degradation mechanisms.

## Figures and Tables

**Table 1:** PUR objects investigated and collected samples.

Artwork	Author, Date	Sample	Description
Reference	Carlo de Incontrera, 2020	REF	Unpainted
Rainbow (*Arcobaleno*)	Miela Reina, 1960s	R_1	Purple
		R_2	Blue
		R_3	Green
		R_4	Yellow
		R_5	Orange
		R_6	Red
		R_7	Dark orange
Parachutist (*Paracadutista*)	Miela Reina, 1960s	PAR_1	Unpainted
Scarf (*Sciarpa*)	Enzo Cogno, 1970s	SCA_1	Unpainted
		SCA_2	Blue
		SCA_3	Orange
		SCA_4	Blue
Scissors (*Forbici*)	Enzo Cogno, 1970s	SCI_1	Unpainted
		SCI_2	Unpainted, glued
Arrow (*Freccia*)	Enzo Cogno, 1970s	AR_1	Unpainted
		AR_2	Blue
		AR_3	Red, glued
Dragon (*Drago*)	Enzo Cogno, 1970s	DR_1	Unpainted
		DR_2	Dark orange
		DR_3	Red
		DR_4	Unpainted
		DR_5	Green
Big vibrating character (*Grande personaggio vibrante*)	Enzo Cogno, 1970s	BVC_1	Unpainted
		BVC_2	Yellow
		BVC_3	Red, glued
Glove (*Guanto*)	Enzo Cogno, 1970s	GL_1	Red
		GL_2	Blue
Tree (*Albero*)	Enzo Cogno, 1970s	TR_1	Red
Envelope (*Busta*)	Carlo de Incontrera, 2020	ENV_1	Unpainted
		ENV_2	Blue

## Data Availability

Data will be made available on request.

## Supplementary Materials

The following supporting information can be downloaded at: https://www.mdpi.com/article/10.3390/polym15092111/s1, Figure S1: infrared spectrum of PUR-ET substrate; Table S1: PUR-ET tentative of attributions in REF sample according to the literature; Table S2: Compounds identified by Py(cryo)-GC/MS in the samples from the different artworks; Table S3: Binder’s identification on selected artworks by μ-FTIR and * by Py(cryo)-GC/MS; Table S4: Pigments identified Raman spectroscopy measurements; Figure S2: μ-Raman spectra acquired on the different color of Rainbow; Figure S3: OM images acquired at 63× on the selected sample; Figure S4: Secondary electrons SEM images on the selected sample; Table S5: average cell strut evaluated by the analysis of SEM images.
