# Supplementary material for: Preserving the Ephemeral: A Micro-Invasive Study on a Set of Polyurethane Scenic Objects from the 1960s and 1970s
Source: Polymers (Basel). 2023 Apr 28;15(9):2111. doi: 10.3390/polym15092111 (PMC10181275; doi:10.3390/polym15092111)
Supplement: Supplementary file 1 [file polymers-15-02111-s001.zip › polymers-2337744-supplementary.pdf]

Supplementary Materials

Preserving the ephemeral: a micro-invasive study on a set of polyurethane scenic objects from the *1960s* and *1970s*.

Rosa Costantini [1], Luca Nodari [1,*], Jacopo La Nasa [2], Francesca Modugno [2], Lucia Bonasera [2], Sara Rago [3], Alfonso Zoleo [4], Stefano Legnaioli [5] and Patrizia Tomasin [1]

[1] Institute of Condensed Matter Chemistry and Technologies for Energy, National Research Council, (ICMATE-CNR), Corso Stati Uniti 4, 35127, Padua, Italy; rosa.costantini@icmate.cnr.it; luca.nodari@cnr.it, patrizia.tomasin@cnr.it
[2] Department of Chemistry and Industrial Chemistry, University of Pisa, Via Giuseppe Moruzzi 13, 56124 Pisa, Italy; jacopo.lanasa@unipi.it, francesca.modugno@unipi.it, l.bonasera@studenti.unipi.it
[3] Department of Cultural Heritage: Archaeology and History of Art, Cinema and Music, University of Padova, Piazza Capitaniato 7, 35139 Padova, Italy; sara.rago@studenti.unipd.it
[4] Department of Chemical Sciences, University of Padova, Via Marzolo 1, 35131 Padova, Italy; alfonso.zoleo@unipd.it
[5] Institute of Chemistry of OrganoMetallic Compounds, National Research Council, (ICCOM-CNR), Via Moruzzi 1, 56124, Pisa, Italy; stefano.leganioli@cnr.it
* Correspondence: luca.nodari@cnr.it

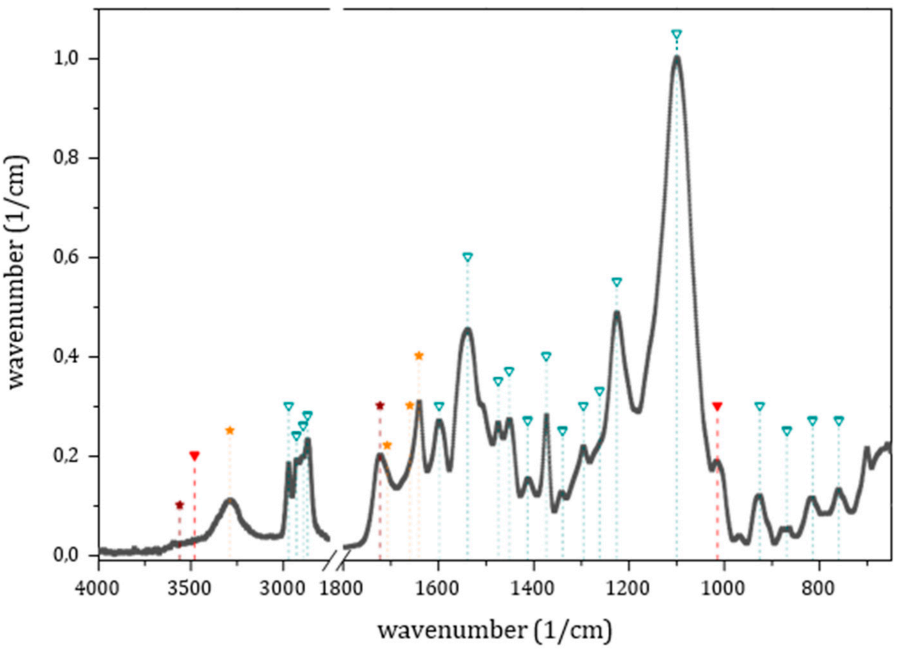

Figure S1. µ-FTIR spectrum or Reference substrate. Dark cyan triangles stand for the PUR attributions: yellow and dark red stars highlight the signals due to non H-bonded absorptions and H-bonded absorptions respectively; red triangles highlight the signals due to photo-oxidation products.

Table S1. PUR-ET tentative of attributions in REF sample according to literature [La Nasa, J.; Biale, G.; Ferriani, B.; Colombini, M.P.; Modugno, F. A pyrolysis approach for characterizing and assessing degradation of polyurethane foam in cultural heritage objects. Journal of Analytical and Applied Pyrolysis 2018, 134, 562-572, https://doi.org/10.1016/j.jaap.2018.08.004; França de Sá, S.; Ferreira, J.L.; Pombo Cardoso, I.; Macedo, R.; Ramos, A.M. Shedding new light on polyurethane degradation: Assessing foams condition in design objects. Polymer Degradation and Stability 2017, 144, 354-365, https://doi.org/10.1016/j.polymdegradstab.2017.08.028]. Red lines highlight the absorptions affected by the alteration process and in blue the absorption due the insurgence of new signals, ascribable to the R-OH formation.

Position	Attribution	Position	Attribution
**3561±4; b, w**	ν(N-H) non H-bonded	**1452±4; m**	δ_a(C-H_3)
**3480±4; sh**	ν(O-H) H-bonded	**1413±4; w**	Isocyanurate ring
**3289±4; m**	ν(N-H) H-bonded	**1374±4; m**	δ_s(C-H_3)
**3067±4; vw**	ν(C-H) in benzene ring	**1340±4; w**	ω(C-H_2)
**2971±4, s**	ν_a(C-H_3)	**1296±4; w**	ν(C-N)
**2929±4, s**	ν_a(C-H_2)	**1262±4; sh**	ν(O=C-N-H)
**2893±4, s**	ν(C-H)	**1226±4; s**	ν(C-N)
**2869±4, s**	ν_s(C-H_3)	**1100±4; vs**	ν_s(C-O-C)
**1723±4; m**	ν(C=O) urethane, free	**1015±4; w**	ν(C-O-H)
**1708±4; sh**	ν(C=O) urethane, loosely H-bonded	**926±4; w**	ρ(C-H_3)
**1660±4; sh**	ν(C=O) monodentate urea, disordered H-bond	**869±4; w**	ω(C-H) benzene ring
**1643±4; m**	ν(C=O) urea H-bonded	**815±4; w**	ω(C-H) benzene ring
**1599±4; m**	ν(C-H) in benzene rings	**760±4; w**	ω(C-H) benzene ring
**1535±4; s**	δ(O=C-N-H)	**702±4; w**	δ(C-H) benzene ring
**1475±4; m**	δ(C-H_2)		

Table S2. Compounds identified by Py(cryo)-GC/MS in the samples from the different artworks.

Nº	Peak identification	Main ions (m/z)
1	Acrolein	67, 56, 37, 29
2	Isoprene	68, 67, 53
3	Propanal	58, 57
4	Toluene	92, 91, 65, 63
5	Ethyl isothiocyanate	87, 72, 59
6	Ethylbenzene	106, 91, 77, 65
7	1-isopropoxy propan-2-one	63, 59, 58
8	1,4-pentadiene	67, 53
9	2-ethyl-1-hexene	112, 70, 55
10	Styrene	104, 103, 78
11	2-methylstyrene	118, 117, 103, 91, 78, 63
12	2-methylene-4,6-diphenyl hexanitrile	128, 117, 91, 77, 65
13	4,6-diphenylept-6-ennitrile	144, 118, 115, 91, 77
14	2-phenylethyl-4-phenylpent-4-enenitrile	170, 156, 142, 118, 115, 105, 91, 77
15	Toluene diisocyanate (2,4 and 2,6 isomers)	174, 145, 132, 118, 91, 76
16	Tripropylene glycol	103, 59
17	Dipropylene glycol	73, 59, 51
18	Erucamide	126, 112, 97, 62, 59
19	Squalene	137, 121, 95, 81, 69
20	Oleic acid	111, 97, 83, 69, 55
21	Cholesterol	329, 129, 105, 95,81
22	Diethyl phthalate	177, 149, 121, 105, 93, 76, 75
23	4-methyl-3-hexanol	87, 69, 59
24	2-ethylhexanol	70, 57, 55
25	Bis(2-ethylhexyl) phthalate (DEHP)	279, 167, 149, 113, 71, 57
26	Isobutyl methacrylate (iBMA)	87, 69, 56
27	Chloroprene	90, 88, 62, 53
28	1-chloro-4-(1-chlorovinyl)cyclohexene	176, 141, 105, 90, 88, 65
29	Unidentified	239, 193, 179, 165, 152, 115, 91, 79
30	Unidentified	254, 239, 191, 178, 165, 152, 141, 128, 115
31	3-methylphenol	108, 107, 90, 79, 77, 63
32	4-tert-butylphenol	150, 135, 107, 95, 91, 77, 65
33	4-tert-butil-2-methylphenol	164, 149, 121, 91, 77
34	2,4-dimethyl-6-tert-butylphenol	178, 163, 135, 91, 77
35	5-methyl-2,4-diisopropylphenol	192, 177, 135, 115, 91, 77
36	Benzene	78, 74, 51
37	Acetic acid	60
38	Xylene and isomers	106, 105, 91, 77
39	1-heptene	98, 70, 56
40	1-decene	140, 111, 97, 83, 70, 55
41	1-undecene	154, 126, 111, 97, 83, 70, 55
42	2-methyl-1-undecene	168, 140, 83, 69, 56
43	1-tetradecene	196, 125, 111, 97, 83, 70, 55
44	1-heptadecene	238, 125, 111, 97, 83, 69, 55
45	1-octadecene	252, 125, 111, 97, 83, 69, 55
46	1-nonadecene	266, 139, 125, 11, 97, 83, 69, 55
47	1-eicosene	280, 139, 125, 111, 96, 83, 69, 57

Table S3. Binder's identification on selected artworks by μ-FTIR and * by Py(cryo)-GC/MS

Artworks	Sample	Polyvinyl acetate	Acryl-based
Rainbow	*R_1. R_2, R_3, R_4, R_5, R_6, R_7*	✓	
Scarf	*SCA_2, SCA_3, SCA_4*	✓	
Arrow	*AR_3*	✓ *	
Big Vibrating Character	*BVC_2, BVC_3*	✓ *	
Glove	*GL-1*	✓	
	GL-2		✓
Tree	*TR_1*	✓	
Envelope	*ENV_2*		✓ *

Table S4. Pigments identified Raman spectroscopy measurements. Vs: very strong, s: strong, m: medium.

Sample	Colour	Raman modes (cm^{-1})	Pigment
**R_1**	Blue	257, 547(vs), 805, 1096(m), 1355, 1643, 1801, 1927	Ultramarine ($Na_{8-10}Al_6Si_6O_{24}S_{2-4}$)
**R_2**	Indigo	258, 548(vs), 807, 1096(m), 1354, 1644	Ultramarine ($Na_{8-10}Al_6Si_6O_{24}S_{2-4}$)
**R_3**	Green	200, 292, 640, 683(vs), 705(m), 739(s), 777(s), 817, 976(m), 1080(m), 1213(vs), 1281(s), 1336(s), 1388, 1444(s), 1537(vs)	Phthalocyanine
**R_4**	Yellow	342, 444, 618, 1065, 1135, 1247, 1274(vs), 1315(vs), 1389(s), 1478, 1508, 1548(m), 1591(vs)	PY16 C.I. 20040, (Disazo pigment, Bisacetoacetarylide)
**R_5**	Orange	263, 342, 456, 623, 918, 985(s), 1141, 1253(s), 1291(m), 1334(m), 1400(s), 1596(vs)	PY83 C.I. 21108, (Disazo pigment, Diarylide)
**R_6**	Red	467, 527(m), 725, 746, 812, 957, 1060(m), 1110(m), 1161(m), 1230(s), 1282(s), 1315, 1359(vs), 1484(s), 1552(m), 1579(vs)	PR112 C.I. 12370, (Monoazo pigment, Naphthol AS)
**R_7**	Dark orange	345(s), 442, 454, 527(m), 615, 662, 680(s), 725, 746, 811, 918, 962(s), 1060(s), 1108(s), 1160(vs), 1254(vs)	PY83 C.I. 21108, (Disazo pigment, Diarylide)
		1281(vs), 1333(m), 1355(vs), 1374(s), 1390(vs), 1460s, 1484(s), 1552(s), 1596(vs)	PR112 C.I. 12370, (Monoazo pigment, Naphthol AS)
**AR_2**	Blue	2145, 1187, 1351	Prussian blue ($Fe_4[Fe(CN)_6]_3$)
**AR_3**	Red	338(m), 567(m), 666, 965, 1045(m), 1110, 1157(m), 1235, 1257, 1280(m), 1364(vs), 1423(m), 1448, 1490(m), 1553(m), 1583(vs), 1606	PR9 C.I. 12460, (Monoazo pigment, Naphtol AS)
**BVC_2**	Yellow	460(m), 785, 999, 1137(m), 1178, 1192, 1215(m), 1254(m), 1310(s), 1322(m), 1386(m), 1485(s), 1533, 1621(s), 1670	PY1 C.I. 11680, (Monoazo pigment, acetoacetic arylide)
**GL_1**	Red	338(m), 567(m), 666, 965, 1045(m), 1110, 1157(m), 1235, 1257, 1280(m), 1364(vs), 1423(m), 1448, 1490(m), 1553(m), 1583(vs), 1606	PR9 C.I. 12460, (Monoazo pigment, Naphtol AS)
**GL_2**	Blue	548(vs), 802, 1096s, 1359, 1647(m)	Ultramarine ($Na_{8-10}Al_6Si_6O_{24}S_{2-4}$)
**TR_1**	Red	345(m), 706, 752, 782, 865(m), 926, 1102(m), 1358(m), 1614	PR122/PV19 (Polyciclic pigment, Quinacridone)
**ENV_2**	Blue	548(vs), 802, 1096(s), 1359, 1647(m)	Ultramarine ($Na_{8-10}Al_6Si_6O_{24}S_{2-4}$)

Sample	μ-Raman spectrum / peaks	Identification
R_1	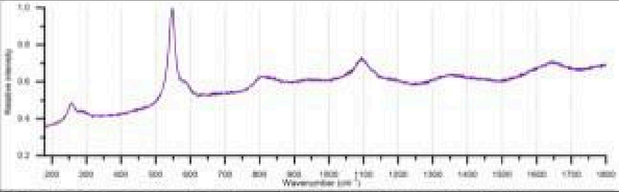<br>257, 547vs, 805, 1096m, 1355, 1643, 1801, 1927	*Ultramarine*
R_2	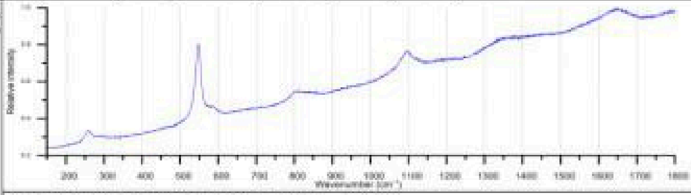<br>258, 548vs, 807, 1096m, 1354, 1644	*Ultramarine*
R_3	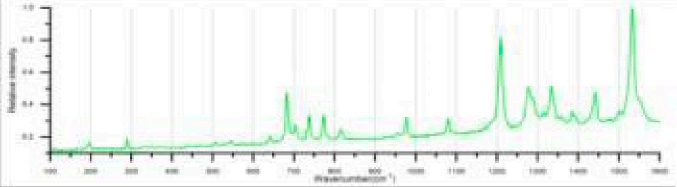<br>200, 292, 640, 683vs, 705m, 739s, 777s, 817, 976m, 1080m, 1213vs, 1281s, 1336s, 1388, 1444s, 1537vs	*Phthalocyanine*
R_4	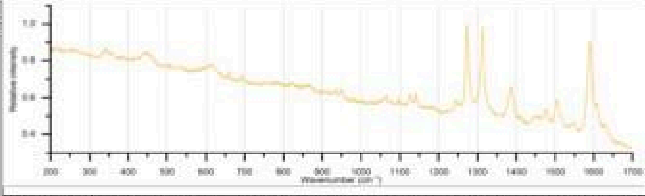<br>342, 444, 618, 1065, 1135, 1247, 1274vs, 1315vs, 1389s, 1478, 1508, 1548m, 1591vs	*PY16 C.I. 20040, Disazopigment, Bisacetoacetarylide*
R_5	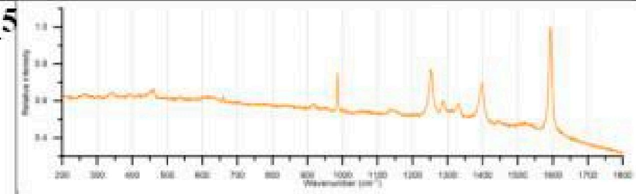<br>263, 342, 456, 623, 918, 985s, 1141, 1253s, 1291m, 1334m, 1400s, 1596vs	*PY83 C.I. 21108, Disazopigment, Diarylide*
R_6	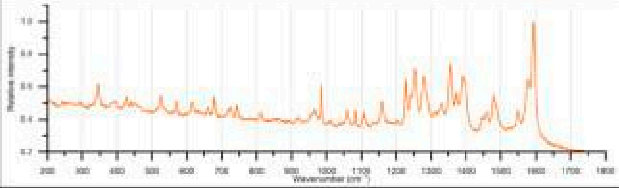<br>345s, 442, 454, 527m, 615, 662, 680s, 725, 746, 811, 918, 962s, 1060s, 1108s, 1160vs, 1254vs, 1281vs, 1333m, 1355vs, 1374s, 1390vs, 1460s, 1484s, 1552s, 1596vs	*PR112 C.I. 12370, Monoazopigment, Naphthol AS*
R_7	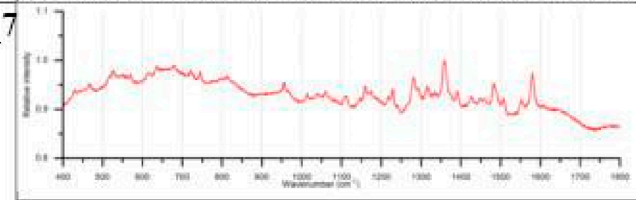<br>467, 527m, 725, 746, 812, 957, 1060m, 1110m, 1161m, 1230s, 1282s, 1315, 1359vs, 1484s, 1552m, 1579vs	*PR112 C.I. 12370, Monoazopigment, Naphthol AS*<br>*PY83 C.I. 21108, Disazopigment, Diarylide*

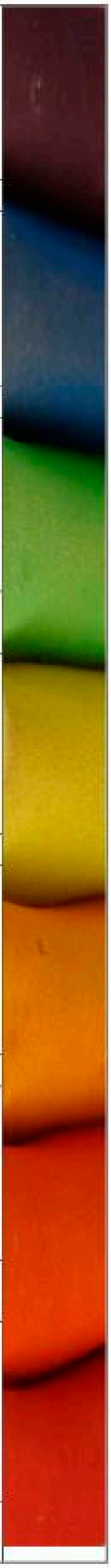

Figure S2. μ-Raman spectra acquired on the different color of Rainbow. The spectra are presented together with the identifying peaks.

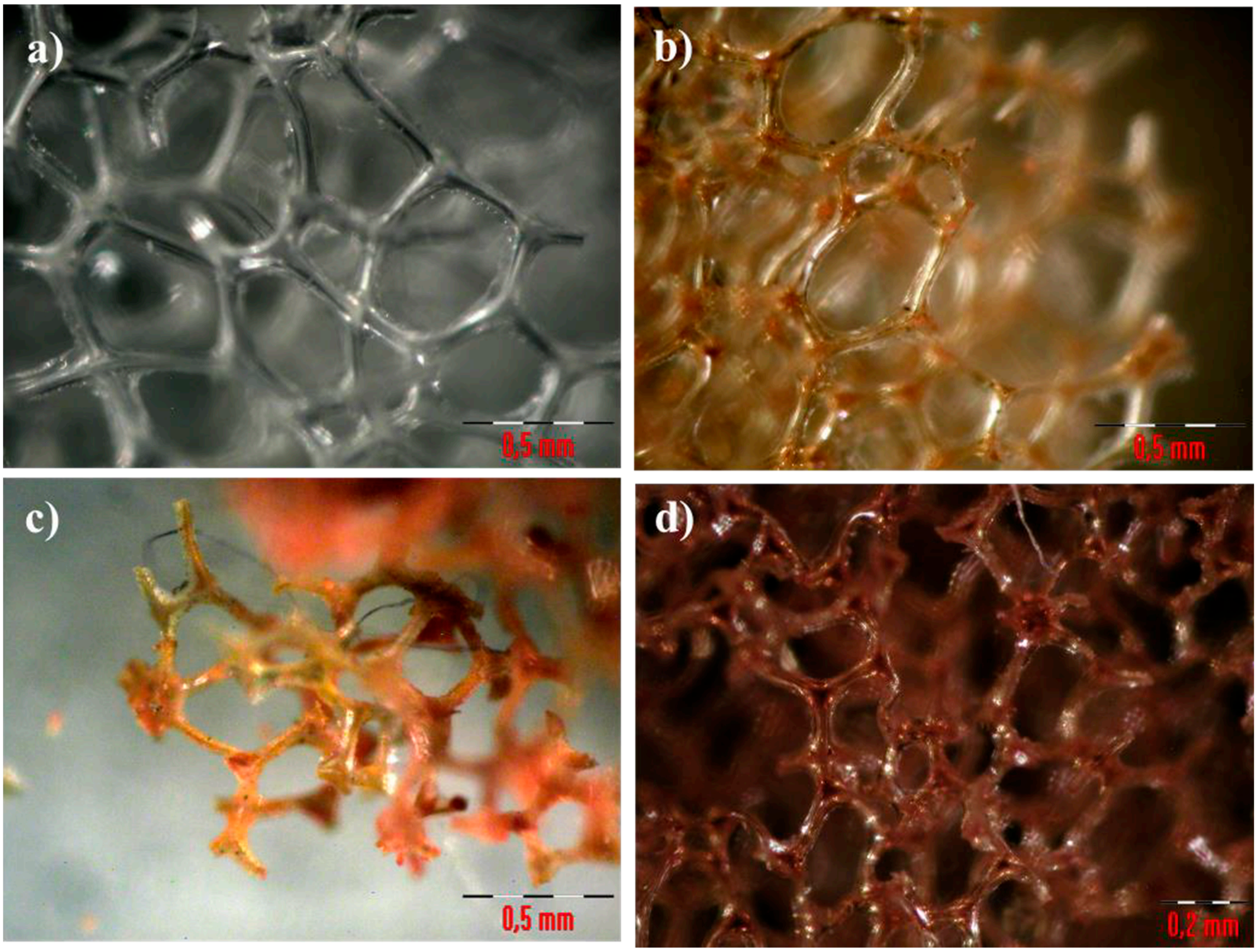

Figure S3. OM images acquired at 63X on: a) REF, b) Big vibrating character, c) Rainbow and d) Parachutist.

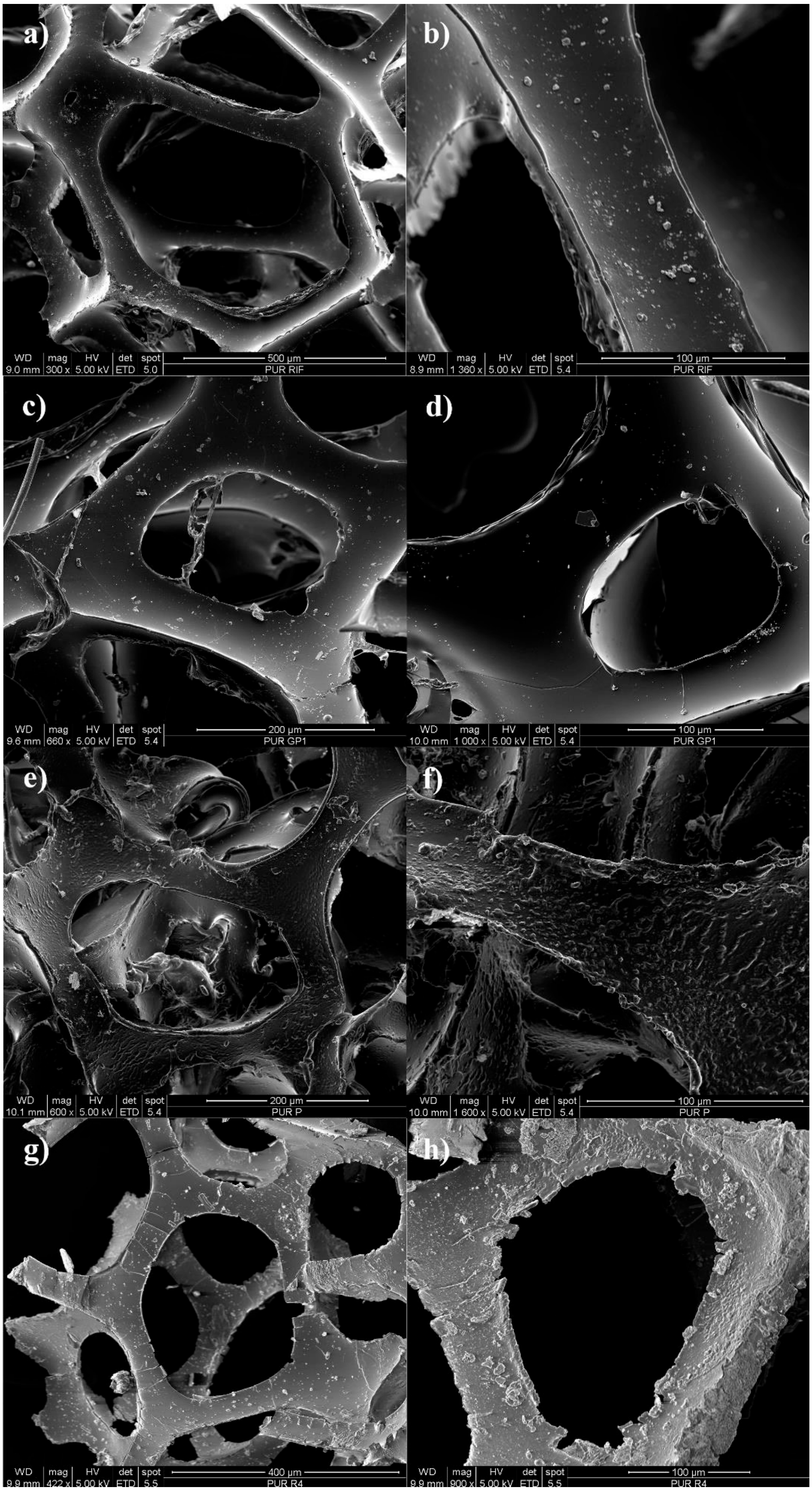

Figure S4. Secondary electrons SEM images of samples from: REF (a-b); *Big Vibrating Character* (c-d); *Parachutist* (e-f); *Rainbow* (g-h)

Table S5. average cell strut evaluated by the analysis of SEM images. The average cell strut was calculated over at least 10 measurements on different cells. We assume an error of 5% of the average value.

Sample	Author	Period	Length (μm)
**REF**	De Incontrera	2020	71±4
**Env_1**	De Incontrera	2020	67±4
**AR_1**	Cogno	1970s	63±3
**BVC_1**	Cogno	1970s	62±3
**SCA_1**	Cogno	1970s	62±3
**PAR_1**	Reina	1960s	55±3
**R_4**	Reina	1960s	55±3
